# Oxidative stress in pigs and opportunities with selenium-containing amino acids

**DOI:** 10.1007/s44154-026-00328-y

**Published:** 2026-08-10

**Authors:** Sung Woo Kim, Nathalia de Oliveira Telesca Camargo, Olivier Merdy, Tadele Kiros, Yan Zhao

**Affiliations:** 1 Department of Animal Science, North Carolina State University, Raleigh, NC USA; 2 Federal University of Rio Grande Do Sul, Porto Alegre, RS Brazil; 3 Phileo By Lesaffre, Marcq-en-Barœul, France; 4 Phileo By Lesaffre, North America, Milwaukee, WI USA; 5 College of Animal Science, Shanxi Agricultural University, Jinzhong, China

**Keywords:** Oxidative stress, Pigs, Selenium, Selenium enriched yeast, Selenocysteine, Selenomethionine

## Abstract

Modern pig farming practices, characterized by highly-productive and fast-growing genetics, early weaning, and uncontrolled factors such as heat stress and various nutritional challenges, impose cumulative stress on the animal. Elevated concentrations of reactive oxygen species within cells can compromise cellular function and decrease reproductive performance of breeding stock and the growth of progeny up to market age, and deteriorate pork quality, ultimately causing economic losses. Antioxidants and micronutrients can be supplemented in animal feed to alleviated the adverse impact of oxidative stress on pig health and production performance. Selenium is an essential micronutrient required for an efficient antioxidant defense system. Se-enriched yeast serves as a natural source of organice selenium. Its effectiveness in improving selenium (Se) assimilation and oxidative status in modern pigs, especially under intensee stress conditions, has been well documented. This review aims to address oxidative stress challenges encountered by pigs during commercial production and elaborate on the capacity of organic Se to mitigate oxidative stress in pigs. Se-enriched yeast provides biologically and nutritionally important amounts of SeMet and SeCys. The review summarizes evidence that, compared wtih inorganic Se, dietary Se-enriched yeast effectively alleviates oxidative stress in pigs while enhancing production performance and pork quality.

## Introduction

### Reactive oxygen species

Animals, including pigs, need energy to sustain life and grow. Energy is obtained from cellular nutrient metabolism, which requires oxygen for the oxidative process, mainly in the mitochondria. This process generates reactive oxygen species (ROS), including superoxide (O_2_^•−^), nitric oxide radical (NO^•^), and hydrogen peroxide (H_2_O_2_). Reactive oxygen species are generated as a part of normal function for maintenance, growth, and reproduction of animals. Still, they are also particularly known for their undesirable actions, causing damage to various biomolecules. These damaging effects on cells are even part of the defense mechanisms of animals to protect the body. Indeed, in a specific immune status, such as a pathogenic challenge condition, immune cells such as macrophages and neutrophils also produce large amounts of ROS called ‘oxidative burst’ or ‘respiratory burst’, to cause oxidative damage to pathogens nearby.

### Antioxidant systems against cellular oxidative stress

Upon the production of ROS as a part of normal cellular functions and immune reactions, cells have redox systems with various antioxidants to remove ROS continuously. These defense mechanisms are mainly composed of antioxidant enzymes and nonenzymatic antioxidants. Typical cellular antioxidants include glutathione (GSH), melatonin, ascorbic acid, carotenoids, tocopherols, polyphenols, thiols, and uric acid, which are synthesized in a cell and obtained from dietary sources. Antioxidant enzymes mainly include superoxide dismutase (SOD), glutathione peroxidase (GSH-Px), and catalase. In a normal condition, healthy cells will effectively remove ROS using these antioxidants with enzymes. The increase in ROS production by the organism consumes neutralizing antioxidant molecules and enhances the production of antioxidant enzymes and molecules. When the ability to produce or obtain antioxidants is depleted or when the production of ROS exceeds the capacity of available antioxidants, the redox balance (the balance between ROS and antioxidants) is disturbed, resulting in oxidative stress and damage to the cell and the tissues (Kim et al. 2011). Oxidative stress is then defined as "an imbalance between oxidants and antioxidants in favor of the oxidants, leading to molecular damages".

### Cellular damage due to oxidative stress

Unsaturated fatty acids are the primary target of peroxidation by ROS. Peroxidation of unsaturated fatty acids propagates lipid oxidative, disrupting membrane integrity and leading to cell death. Fatty acid peroxidation results in the production of malondialdehyde (MDA). The MDA concentration in plasma or tissue can effectively indicate the degree of oxidative damage to lipids (Shen et al. 2012; Zhao et al. 2013; Lipiński et al. 2019). Due to their lipophilic property, tocopherols (one of the vitamin E) are effective antioxidants to prevent fatty acid peroxidation. Other antioxidants and antioxidative enzymes, including selenoproteins, are also involved in the process. Cellular proteins, including enzymes and membrane proteins, are altered and damaged by ROS. The ROS can cause the hydrolysis of peptide bonds, specifically at proline and histidine degrading proteins. Additionally, ROS can cause the production of disulfide bonds between cysteine residues and oxidation of methionine to methionine sulfoxide, altering the active site of proteins and their functions. The ROS can also cause the carbonylation of proteins modifying the side chain of lysine, arginine, proline, threonine, and cysteine (Levine 2002; Dalle-Donne et al. 2003). Carbonylation of a protein is an irreversible process that causes the degradation of proteins. The carbonyl concentration in the plasma or tissue can effectively indicate the degree of oxidative damage to protein (Shen et al. 2014; Tonni et al. 2014; Duarte and Kim 2024). Glutathione, together with GSH-Px, is an effective antioxidant to prevent oxidative damage to cellular proteins. Other antioxidants and antioxidative enzymes are also involved in the process.

Finally, ROS can cause alteration to the base of the nucleic acids in the mitochondria and the nucleus, resulting in genetic mutation, dysfunction, and cell death. The oxidation of nucleic acids produces altered bases including 8-hydroxy deoxy-guanosine, thymidine glycol, and uric acid. The 8-hydroxy deoxy-guanosine concentration in plasma or tissue can be an effective indicator of the degree of oxidative damage to DNA (Collins et al. 2005; Berchieri‐Ronchi et al. 2010; Weaver and Kim 2014). Oxidative stress plays a significant role in pigs’ productivity. It is a primary factor in impaired performance and health at the pig’s various stages of development. By measuring oxidative damage markers in pigs, researchers can assess the extent of stress and design dietary interventions to mitigate it. This article aims to explore the oxidative stress challenges pigs face during the production process and understand the role of organic Se providing oxidative stress control in pigs.

## Oxidative stress in pigs

This section discusses how oxidative stress affects different stages of the pigs life cycle, including nursery pigs, finishing pigs, sows, and boars. The factors that contribute to oxidative stress, its consequences on both intestinal and overall health, and the overall production performance are explored. Furthermore, potential approaches to alleviate oxidative stress through improved nutritional strategies are discussed.

### Sows and their progeny

Sows, particularly during gestation and lactation, are highly vulnerable to oxidative stress (Berchieri‐Ronchi et al. 2010) due to the significant metabolic demands required to support both fetal development (McPherson et al. 2004; Ji et al. 2006; Lee and Roh 2023) and milk production (Kim et al. 1999, 2009). This process is characterized by the accumulation of active oxygen, including O_2_ and H_2_O_2_, produced by the placenta and mammary glands (Casanueva and Viteri 2003). This accumulation impairs reproductive and lactation performance. They even tend not to fully recover until the weaning period (Berchieri‐Ronchi et al. 2010).

Various factors can exacerbate this susceptibility, including nutritional deficiencies, feed contaminated with mycotoxins, immune challenges, and poor environmental conditions such as elevated temperatures and overcrowded housing during these critical periods (Lipiński et al. 2019; Zhao and Kim 2020; Papatsiros et al. 2023). Specifically, heat stress is one of major challenges for sows and finishing pigs. Heat stress induces an increase in electronic leakage from the mitochondria causing generation of ROS which leads to oxidative stress (Belhadj Slimen et al. 2014) and the production of heat shock proteins. Elevated oxidative stress is shown to negatively affect reproductive processes including oocyte maturation, embryo development, and pregnancy (Renaudeau and Noblet 2001; Karowicz-Bilinska et al. 2002).

Inadequate dietary intake of antioxidants enhances the problem, making it more challenging for sows to regulate ROS and maintain oxidative balance (Li et al. 2021). Oxidative stress can impair ovarian function and disrupt embryo development, resulting in irregular estrous cycles, lower conception rates, and increased miscarriages or fetal loss (Guerin et al. 2001; Zhao et al. 2013). Newborn piglets are particularly susceptible to oxidative stress at birth due to an immature antioxidant system (Yin et al. 2013). Thus, fetal development and growth processes are susceptible to oxidative stress and may continuously have a negative effect on the progeny. During lactation, oxidative stress was found to decrease milk production and impair milk quality, therefore, affecting piglet growth, health, and survival (Kim et al. 2013).

### Nursery pigs

In pig production, pigs are weaned at an early age, before intestinal maturation is complete. Upon weaning from sows’ milk, pigs are fed solid diets based on cereals, legume seed meals, milk coproducts, and animal proteins. Even though these diets are nutritionally well-balanced, meeting the pigs’ requirements, several compounds in these diets would cause changes in intestinal microbiota and mucosa immune responses which lead to oxidative stress conditions due to massive production of ROS from the intestinal immune cells (Zheng et al. 2021).

Dietary challenges causing intestinal microbial disturbance have been observed. These microbial changes are shown to cause mucosal immune responses in the small intestine. Dietary and pathogenic challenges could also directly cause mucosal immune responses. Soluble fiber, for example, can alter microbial composition, increasing the proportion of potentially harmful bacteria (Baker et al. 2024; Choi et al. 2024; Jang et al. 2024) and the intestinal mucosa would recognize these changes by pattern recognition receptors (Duarte et al. 2024a; Duarte and Kim 2024). In turn, this signals the intestinal immune cells to be activated (Holanda and Kim 2022; Moita et al. 2022; Xu et al. 2022) and increases the production of ROS, causing oxidative damage to the inte*stinal mucosa. Allergens and antigens in the diet also directly cause the mucosal immune reac*tion (Deng et al. 2023a, b), resulting in oxidative tissue damage in the small intestine (Deng et al. 2022; Duarte et al. 2024b). During the nursery period, the maintenance of a high antioxidant capacity correlates with better growth performance under commercial conditions (Buchet et al. 2017; Long et al. 2021).

### Finishing pigs

In this fast-growing stage, oxidative stress is commonly induced by environmental, management, and nutritional factors. High ambient temperatures, particularly when coupled with humidity, significantly contribute to oxidative stress in finishing pigs due to their sensitivity to heat (Cui et al. 2016). Additionally, transportation and overcrowded housing conditions exacerbate their susceptibility to oxidative stress (Marco-Ramell et al. 2011; Zhang et al. 2015). Nutritional factors also play a crucial role in influencing oxidative stress levels. For example, feed that is peroxidized, contaminated with mycotoxins, or deficient in essential antioxidants like vitamin E and selenium (Se) exacerbates the oxidative burden on finishing pigs (Frame et al. 2020; Hao et al. 2021; Holanda and Kim 2021).

The impact of oxidative stress on intestinal health in finishing pigs is significant, including impaired nutrient absorption and increased risk of gastrointestinal diseases (Overholt et al. 2018). A study showed that a low-fiber diet (3.4%) contributed to elevated oxidative stress in the jejunum, as indicated by increased MDA concentration compared with a high-fiber diet (6.3%). This change necessitated greater production of antioxidant substances to cope with oxidative stress in the intestine (Jin et al. 2022). Research has also found that oxidative stress, induced by heat stress or dietary factors, could damage a pig’s intestinal epithelial cells, impairing the mucosal barrier functions in the intestine (Fan et al. 2020; Xia et al. 2022). These conditions disrupt intestinal microbiota, promote intestinal inflammation, and impair nutrient absorption (Xia et al. 2022).

Systemically, oxidative stress reduces growth performance, as energy and nutrients are redirected from growth processes to counter oxidative damage (Aguilar et al. 2008). Prolonged oxidative stress could weaken the immune system, making pigs more vulnerable to infections (Cui et al. 2016).

Additionally, oxidative stress was found to have negative effects on pork quality, including undesirable changes in meat color, shelf life, texture, and tenderness (Lu et al. 2014; Zou et al. 2016; Guo et al. 2021).

### Boars

In modern production systems, high-frequency breeding can induce physiological stress in boars, increasing ROS production (Lopez Rodriguez et al. 2017). Nutritional imbalances, for example, when a diet is deficient in antioxidants, could also lead to oxidative stress (Brambilla et al. 2002; Liu et al. 2017). Oxidative stress was found to have a significant effect on semen quality. It can cause lipid peroxidation and DNA damage in sperm cells, leading to reduced sperm motility, increased abnormal sperm, and a decline in overall reproductive efficiency (Pilane et al. 2016). These changes can severely affect fertility and, therefore, reduce conception rate and overall reproductive success. Furthermore, oxidative stress weakens the immune system of boars, increasing their susceptibility to infections (Gasso et al. 2016). Boars under oxidative stress might exhibit decreased energy and stamina, ultimately reducing their productivity and longevity within breeding programs. Therefore, controlling oxidative stress is crucial to maintaining boars’ reproductive performance and well-being.

## The antioxidative compounds and systems to fight against oxidative stress in pigs

Under normal conditions, there is a dynamic balance between the antioxidant system and free radicals, which is essential for the survival and health of an organism. Oxidative stress occurs when there is an imbalance between the production of ROS and the body's ability to neutralize them using antioxidants, which can negatively affect animal health, performance, and meat quality. Pigs, especially in modern production systems, are exposed to various stressors like high-density housing, heat, and high metabolic demand for growth or reproduction, which can lead to increased ROS. Nutritional strategies are an effective and economical approach to mitigate oxidative damage and protect cellular integrity. It is also more suitable for the current intensive farming model (Cottrell et al. 2015).

### The antioxidant system

The antioxidant system defense mechanisms are mainly composed of antioxidant enzymes and non-enzymatic antioxidants. Antioxidant enzymes mainly include SOD, GSH-Px, and catalase with different modes of action. The SOD catalyzes the dismutation of two O_2_^•−^ anions to H_2_O_2_ and dioxygen. Catalase neutralizes H_2_O_2_ into water and oxygen. The GSH-Px is a selenoenzyme that reduces both H_2_O_2_ and organic hydroperoxides respectively to water and alcohols, while oxidizing 2 molecules of GSH antioxidant tripeptide into glutathione disulfide.

Nonenzymatic antioxidants mainly include ascorbic acid (vitamin C), a-tocopherol (vitamin E), GSH, carotenoids, flavonoids, and other phytochemicals. Vitamin E is a fat-soluble vitamin that can enter the cell and reduce peroxyl radicals (ROOꞏ), forming organic hydroperoxides and oxidized vitamin E. Vitamin C is an antioxidant that reduces oxidized vitamin E and is itself maintained in a reduced form by GSH. The GSH is a tripeptide that is required to reduce oxidized antioxidants (e.g. vitamin C) to their active form, and which also acts as a cofactor for the antioxidant enzyme GSH-Px. Glutathione disulfide is formed during both processes and is itself reduced by enzymes such as glutathione reductase.

### Multilevel antioxidant defense

The first level prevents the formation of free radicals. It inactivates, catalyzes the removal of precursors of free radicals and includes the three antioxidant enzymes cited above (SOD, CAT, and GSH-Px). This first level of antioxidant defense is insufficient to fully impede the generation of free radicals in the cell. As a result, some radicals escape, initiating peroxidation of lipids and causing damage to cellular protein and DNA.

The second level of the antioxidant system consists of chain-breaking antioxidants like vitamin C, vitamin E, and carotenoids. Lipid peroxidation by free radicals can induce cascade reactions in the cell membrane where a lipid peroxide radical formed by the interaction of a free lipid radical with oxygen destabilizes a lipid nearby, transforming it into a new free lipid radical. The antioxidants from the second line of defense scavenge the free radical, thus interrupting the chain reaction. Glutathione peroxidase is also part of the secondary line of defense since it catalyzes the reduction of lipid peroxides to lipid alcohols, respectively, as well as catalyzing the oxidization of GSH to glutathione disulfide. Glutathione also plays a significant role since it helps oxidized forms of scavenging molecules such as vitamin C to regenerate their active antioxidant capacity.

Finally, the thioredoxin system is also important in the control of oxidative damage of proteins: thioredoxins are molecules that play an essential role in the reduction of oxidized components, such as Cys-Cys disulfide bonds that could impair protein activity. Thioredoxin reductase, a Se-dependent flavoprotein, then reduces oxidized thioredoxin and its homologs. Thioredoxin reductases can also reduce several other low molecular-weight compounds, such as glutathione disulfide, lipid peroxides and hydrogen peroxides (Kieliszek and Błażejak 2016).

### The vital role of selenium in antioxidant defense

Selenium is a vital trace element that plays an indispensable role in maintaining the overall health of animals and humans. Although required in small amounts, Se is crucial for a wide range of biological processes, particularly in protecting the body against oxidative stress. It functions mainly through its incorporation into specific proteins collectively called selenoproteins. There are 25 selenoproteins which are essential for various physiological mechanisms, including antioxidant defense, immune response, and thyroid hormone metabolism.

Selenium is present in these proteins in the form of selenocysteine (SeCys), which plays a specific functional role as a part of the active site in selenoproteins. Thus, the presence of SeCys is essential for the synthesis and function of selenoproteins. Common functions of selenoproteins include redox processes, SeCys synthesis, Se transport, thyroid metabolism, and other functions (Boyington et al. 1997; Kasaikina et al. 2012). At least 16 selenoproteins have antioxidant roles (Pappas et al. 2008). Major contributions in the antioxidant functions are made by the GSH-Px and thioredoxin reductases (Sordillo 2013). External factors are shown to influence the synthesis of selenoproteins. Heat stress as one of key challenges in pig production influence the gene expression and the synthesis of selenoproteins in relation to antioxidant roles (Tang et al. 2019; Liu et al. 2021a) whereas its influence after acute vs chronic conditions may vary remaining unclear. Detailed mechanisms for the impact of heat stress on Se metabolism are not yet well understood, warranting further investigation.

## Metabolism of selenium-containing amino acids

**Fig. 1:**
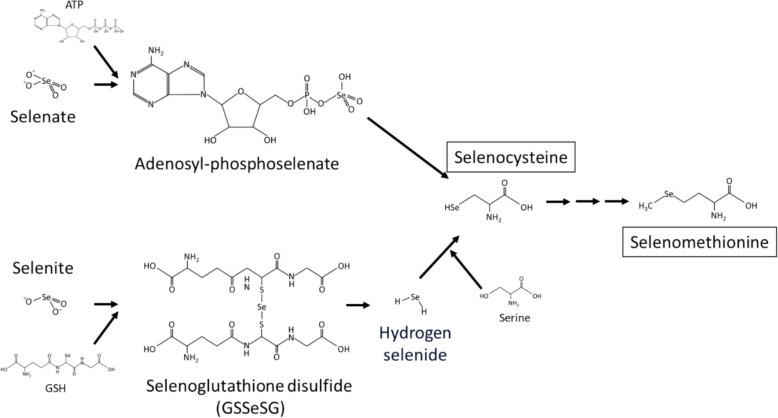
Process of synthesis of selenomethionine and selenocysteine in yeast cells. This figure is designed and drawn by the authors based on concepts from Schrauzer (2000); Papp et al. (2007); Krittaphol et al. (2011); Lima et al. (2018)

All amino acids are synthesized in plant cells, bacterial cells, yeast cells, and fungal cells, whereas animal cells synthesize only a limited number of them. During the process of synthesizing these amino acids, when Se is present, Se can be incorporated in the structure of sulfur-containing amino acids, methionine, and cysteine, instead of sulfur producing SeMet and SeCys (Fig. 1; Schrauzer 2000; Papp et al. 2007; Krittaphol et al. 2011; Lima et al. 2018).

### Utilization of selenium-containing amino acids in protein synthesis

Both SeMet and SeCys can be a part of protein during protein synthesis. This happens not only in the plant cells, bacterial cells, yeast cells, and fungal cells where Se-containing amino acids are synthesized but also in the animal cells if Se-containing amino acids are obtained from food sources.

**Fig. 2:**
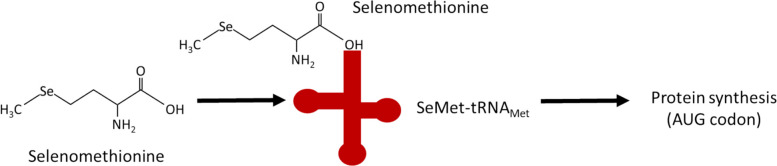
Use of selenomethionine in protein synthesis (Schrauzer 2000; Minich 2022)

Selenomethionine is recognized by the tRNA responsible for the AUG codon, and thus SeMet can replace methionine when it is present. Thus, with the presence of SeMet, a protein can contain SeMet (Fig. 2; Schrauzer 2000; Minich 2022). Therefore, SeMet can be a part of body protein, depending on the half-life of the proteins in which SeMet is included. When cabbage and garlic were grown in the presence of Se, these plants effectively incorporated Se in their tissues (Ip et al. 2000; Seo et al. 2008). In the case of animals, when pigs were fed diets supplemented with SeMet, Se concentrations in the loin were increased (Falk et al. 2018; Rao et al. 2023). It is proposed that these body proteins containing SeMet can be a source of Se when these proteins are degraded.

**Fig. 3:**
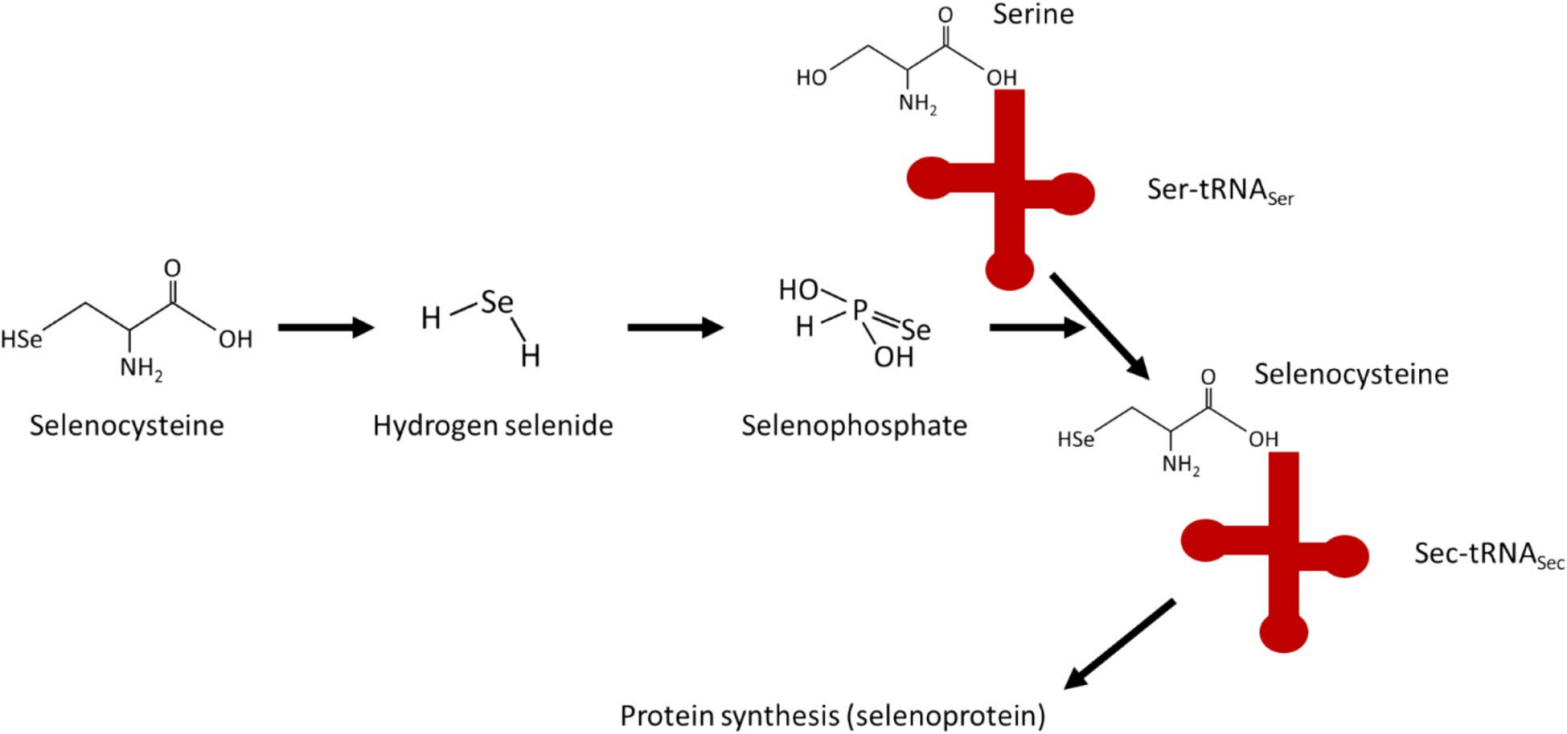
Use of selenocysteine in protein synthesis (Turanov et al. 2013; Hilal et al. 2022; Bierla et al. 2023)

In the case of SeCys, it is encoded by the UGA codon, which is a stop codon in the translation of mRNA. Thus, with the presence of SeCys, a protein will most likely contain one SeCys at the end of its peptide (Fig. 3; Turanov et al. 2013; Hilal et al. 2022; Bierla et al. 2023). Proteins containing SeCys are collectively called selenoproteins. There are 25 selenoproteins identified with varied functions, including oxidoreduction, SeCys synthesis, Se transport, and some unknown others (Moghadaszadeh and Beggs 2006).

### Metabolism of selenium-containing amino acids

#### Trans-selenation pathway for conversion of selenomethionine to selenocysteine

**Fig. 4:**
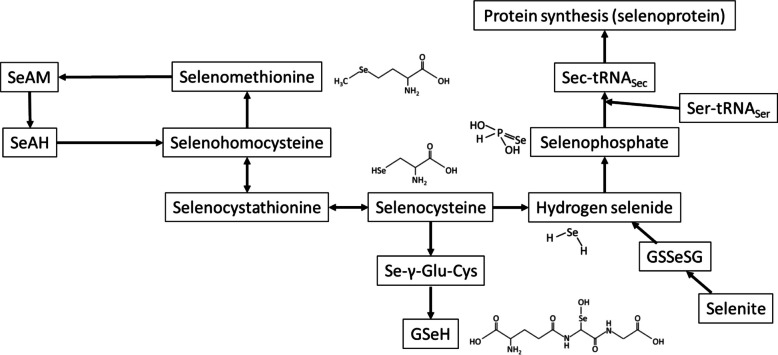
Metabolism of selenium-containing amino acids. This figure is designed and drawn by the authors based on concepts from Burk and Hill (2015); Seale (2019); Bierla et al. (2023)

Proteins undergo continuous turnover. Depending on the types of proteins, half-life varies greatly between a few seconds and a hundred days (Schrauzer 2000; Aguilar et al. 2008). Degradation of SeMet -containing proteins releases SeMet to re-enter the free methionine pool. Either the SeMet is used in the synthesis of other proteins, or it will be metabolized, mostly in the liver, via the trans-selenation pathway yielding SeCys (Esaki et al. 1981). Notably SeMet is first converted into selenohomocysteine. Selenohomocysteine is then converted to selenocystathionine and further metabolized to form SeCys (Fig. 4; Burk and Hill 2015; Seale 2019; Bierla et al. 2023).

#### Selenocysteine as substrate for synthesis of selenoglutathione and selenoproteins

Selenocysteine can be acquired through diet, released after selenoprotein degradation or produced inside cells as a metabolite of SeMet metabolism as described above. With the presence of glutamate and glycine, SeCys can be further metabolized to selenoglutathione. Selenoglutathione is an analog of glutathione with similar functions in scavenging radicals. Steinmann et al. (2008) and Carroll et al. (2020) showed that selenoglutathione possesses greater radical scavenging efficiency than GSH indicating possible animal application under oxidative stress conditions including late gestation and early lactation (Berchieri‐Ronchi et al. 2010; Zhao et al. 2013). When it is not used in the formation of selenoglutathione, SeCys can also be used in the synthesis of selenoproteins.

Considering the metabolism of Se-containing amino acids, the metabolic use of SeMet and SeCys might be uniquely distinct, whilst SeMet and SeCys are interconvertible depending on metabolic status. Selenomethionine is an effective source of Se that can be stored in body protein, whereas SeCys is effectively used in body functions as a part of selenoglutathione and selenoproteins. This uniquely different functional role between SeMet and SeCys implies important application in animal feeding depending on the goal of utilizing the benefits of Se. If the goal is to produce Se-enriched meat, SeMet would provide effective applications. If the goal is to provide Se for antioxidative protection to help the animals under oxidative stress, SeCys would provide effective applications.

#### Incorporation of free selenocysteine into selenoproteins via a unique recycling pathway

**Fig. 5:**
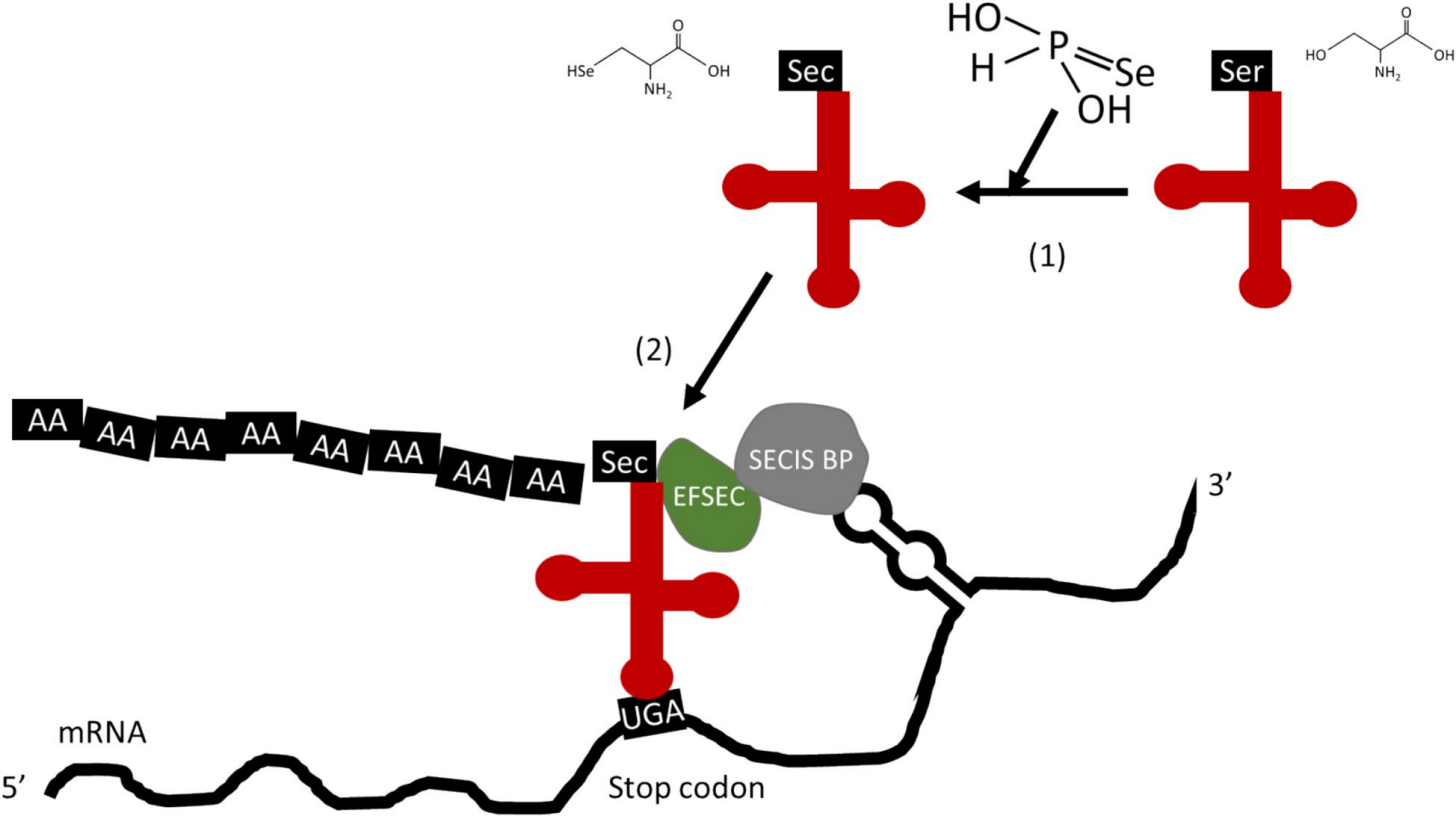
Use of selenocystein in the synthesis of selenoprotein. (1) Serine with tRNA_Ser_ receives selenophosphate by L-seryl-tRNA_Sec_ selenium transferase resulting in the conversion of serine to selenocysteine. (2) Decoding UGA codon requires selenocysteine insertion sequence (SECIS) with SECIS binding protein (SECIS BP) and selenocysteine-specific elongation factor (EFSEC). This figure is designed and drawn by the authors based on concepts from Burk et al. (2001); Labunskyy et al. (2014); Hilal et al. (2022)

Selenocysteine is a critically important part of selenoproteins. Earlier in this review, it was mentioned that SeCys is encoded by the UGA codon in the translation process of mRNA for selenoproteins. This process requires a special stem-loop sequence called SECIS (SeCys insertion sequence) found downstream of the UGA codon (Fig. 5). Importantly, free SeCys needs to be metabolized by SeCys lyase to selenide and alanine to be used in selenoprotein translation. This occurs as the selenophosphate synthetase 2 (SEPHS2) specifically utilizes selenide to produce monoselenophosphate for SeCys biosynthesis. Such a particularity generates a critical recycling step, by which SeCys is broken down to be precisely resynthesized for exclusive use in selenoprotein translation. The SeCys initiating the sequence can be acquired through diets, released after selenoprotein degradation, or produced inside cells as a metabolite of SeMet metabolism via the trans-selenation pathway.

### Selenomethionine in protein synthesis and selenium-enriched meat

When animals consume a diet containing SeMet, it is expected to be used in protein synthesis and thus accreted in the body whereas inorganic source of Se would have a low chance to be incorporated in the body protein. Young and growing animals may have greater efficiency in using SeMet compared with old and mature animals. There are several feeding studies using pigs fed diets containing either an inorganic form of Se, such as sodium selenite (SeO₃⁻^2^) or an organic form of Se including SeMet, which investigated Se accretion in the muscle (Table 1).

**Table 1:** Muscle selenium (Se) concentrations (μg/g) in the muscle of pigs fed diets with different Se sources

Reference	Se source and inclusion (mg/kg)	Body weight (kg) Feeding duration (d)	Se concentration in the muscle (μg/g)
(Rao et al. 2023)	SeO₃⁻^2^ (0.3) Se-enriched yeast (0.3) Selenomethionine (0.3)	13 kg 35 d	SeO₃⁻^2^ (0.81) Se-yeast (0.87) Selenomethionine (1.42)
(Falk et al. 2018)	SeO₃⁻^2^ (0.33) Se-enriched yeast (0.32) Selenomethionine (0.32)	25.6 kg 64 d	SeO₃⁻^2^ (0.30) Se-enriched yeast (0.56) L-Selenomethionine (0.85)
(Jing et al. 2023a)	Selenomethione (0.0) Selenomethione (0.3) Selenomethione (0.6) Selenomethione (0.9)	25 kg 112 d	Selenomethione (0.07) Selenomethione (0.35) Selenomethione (0.65) Selenomethione (0.95)
(Zhan et al. 2007)	SeO₃⁻^2^ (0.3) Selenomethionine (0.3)	60 kg 40 d	SeO₃⁻^2^ (0.14) Selenomethionine (0.35)
(Zhang et al. 2020)	SeO₃⁻^2^ (0.3) Selenomethionine (0.3)	60 kg 50 d	SeO₃⁻^2^ (0.18) Selenomethionine (0.29)
(Mateo et al. 2007)	SeO₃⁻^2^ (0.3) Se-enriched yeast (0.3)	34.4 to 129.9 kg	SeO₃⁻^2^ (0.20) Se-enriched yeast (0.34)
(Mahan and Parrett 1996)	SeO₃⁻^2^ (0.10) SeO₃⁻^2^ (0.30) SeO₃⁻^2^ (0.50) Se-enriched yeast (0.10) Se-enriched yeast (0.30) Se-enriched yeast (0.50)	65.8 to 105.0 kg	SeO₃⁻^2^ (0.101) SeO₃⁻^2^ (0.124) SeO₃⁻^2^ (0.130) Se-enriched yeast (0.143) Se-enriched yeast (0.212) Se-enriched yeast (0.262)
(Mahan et al. 1999)	SeO₃⁻^2^ (0.05) SeO₃⁻^2^ (0.10) SeO₃⁻^2^ (0.20) SeO₃⁻^2^ (0.30) Se-enriched yeast (0.05) Se-enriched yeast (0.10) Se-enriched yeast (0.20) Se-enriched yeast (0.30)	Up to 55 kg	SeO₃⁻^2^ (0.091) SeO₃⁻^2^ (0.103) SeO₃⁻^2^ (0.113) SeO₃⁻^2^ (0.120) Se-enriched yeast (0.113) Se-enriched yeast (0.143) Se-enriched yeast (0.184) Se-enriched yeast (0.206)
(Mahan et al. 1999)	SeO₃⁻^2^ (0.05) SeO₃⁻^2^ (0.10) SeO₃⁻^2^ (0.20) SeO₃⁻^2^ (0.30) Se-enriched yeast (0.05) Se-enriched yeast (0.10) Se-enriched yeast (0.20) Se-enriched yeast (0.30)	Up to 105 kg	SeO₃⁻^2^ (0.106) SeO₃⁻^2^ (0.114) SeO₃⁻^2^ (0.118) SeO₃⁻^2^ (0.124) Se-enriched yeast (0.134) Se-enriched yeast (0.170) Se-enriched yeast (0.249) Se-enriched yeast (0.332)
(Batorska et al. 2017)	SeO₃⁻^2^ (0.3) SeO₃⁻^2^ (0.5) Se-enriched yeast (0.5)	56 kg 42 d	SeO₃⁻^2^ (0.06) SeO₃⁻^2^ (0.06) Se-enriched yeast (0.15)
(Li et al. 2024)	SeO₃⁻^2^ (0.3) Se-enriched yeast (0.3)	9.2 kg 28 d	SeO₃⁻^2^ (0.15) Se-enriched yeast (0.35)
(Kim and Mahan 2001)	SeO₃⁻^2^ (0.05) SeO₃⁻^2^ (0.10) SeO₃⁻^2^ (0.15) SeO₃⁻^2^ (0.20) Se-enriched yeast (0.05) Se-enriched yeast (0.10) Se-enriched yeast (0.15) Se-enriched yeast (0.20)	24.7 kg 84 d	SeO₃⁻^2^ (0.33) SeO₃⁻^2^ (0.28) SeO₃⁻^2^ (0.32) SeO₃⁻^2^ (0.32) Se-enriched yeast (3.38) Se-enriched yeast (5.93) Se-enriched yeast (10.31) Se-enriched yeast (7.65)
(Mahan and Peters 2004)	SeO₃⁻^2^ (0.15) SeO₃⁻^2^ (0.30) Se-enriched yeast (0.15) Se-enriched yeast (0.30)	27.6 kg to 115 kg	SeO₃⁻^2^ (0.27) SeO₃⁻^2^ (0.48) Se-enriched yeast (0.13) Se-enriched yeast (0.13)

Rao et al. (2023) compared three sources of Se in the diets supplemented with SeO₃⁻^2^ (0.3 mg/kg), Se-yeast (0.3 mg/kg), or SeMet (0.3 mg/kg). After 35 d feeding of the diets from 13 kg body weight, pigs had 0.81, 0.87, or 1.42 μg Se in one gram of loin muscle. Falk et al. (2018) conducted a similar study using pigs fed Se diets for 9 weeks from 26 kg body weight (older pigs than pigs used in Rao et al. 2023) showing the loin Se concentrations of 0.30 (SeO₃⁻^2^), 0.56 (Se-yeast), or 0.85 (SeMet) μg/g indicating that the pigs with SeMet had 180% more (*P* < 0.05) Se in the loin than pigs with SeO₃⁻^2^. Jing et al. (2023a) fed pigs at 25 kg body weight for 16 weeks with diets containing different levels of SeMet (0 to 0.9 mg/kg), resulting in 0.9 μg/g Se increase in the muscle. Zhan et al. (2007) compared SeO₃⁻^2^ (0.3 mg/kg) and SeMet (0.3 mg/kg) fed to pigs from 60 kg body weight for 40 d resulting in 0.14 and 0.35 μg/g Se in the muscle, respectively. Zhang et al. (2020) compared SeO₃⁻^2^ (0.3 mg/kg) and SeMet (0.3 mg/kg) fed to pigs from 60 kg body weight for 50 d, resulting in 0.18 and 0.29 μg/g Se in the muscle, respectively. An analysis of these five studies shows that feeding pigs a diet with SeMet effectively increased Se concentration in the muscle compared to the pigs fed a diet with SeO₃⁻^2^. Considering that the fractional synthesis rate of skeletal muscle in growing pigs ranges between 5% to 20% (Rivera-Ferre et al. 2005; Bregendahl et al. 2008; Sarri et al. 2021), feeding a diet with SeMet at least for 20 days should provide sufficient time for Se accretion in the muscle in growing pigs. Muscle Se concentrations after feeding a diet with SeMet for at least 20 days were different depending on the body weight of pigs (or growth stage of pigs). Pigs at an early age (such as 13 kg body weight in Rao et al. 2023) could maintain high Se concentration compared with pigs at an older age (such as 60 kg body weight in Zhan et al. 2007 and Zhang et al. 2020).

Yeast grown with Se could synthesize Se-containing amino acids, SeMet and SeCys, as stated earlier. There are several commercially available non-nutritive feed additives based on yeast containing high Se. These are collectively called Se-yeast or Se-enriched yeast. Typical strains of yeast used in this process include *Saccharomyces cerevisiae* and *Saccharomyces pastorianus*. When yeast uses Se in the synthesis of Se-containing amino acids, it typically produces SeMet and SeCys roughly at 8:1 ratio whereas the exact ratio would be different by various factors such as genetic differences in yeast strains, growth conditions, and Se source and concentration (Aguilar et al. 2008; Kieliszek et al. 2020). Thus, Se from Se-yeast could effectively contribute to muscle Se accretion when fed to the animals, but the efficiency could be various depending on sources.

Mateo et al. (2007) showed that pigs at 34 kg body weight consuming a diet containing Se-yeast (0.3 mg/kg) until 130 kg body weight contained 0.34 μg/g Se in the muscle compared with 0.20 μg/g Se in the muscle of pigs consumed a diet with SeO₃⁻^2^ (0.3 mg/kg). This is 70% increase of Se in the muscle by feeding Se-yeast. Similarly, Rao et al. (2023) showed that pigs with Se-yeast (mg/kg) had 0.56 μg/g Se in the muscle, which is 85% increase compared with 0.30 μg/g Se in the muscle of pigs with SeO₃⁻^2^ (0.3 mg/kg). Mahan and Parrett (1996) and (Mahan et al. 1999, 2005) published dose–response studies comparing both dietary sources of Se (Se-Yeast or inorganic Se) from low inclusion rates (0.05 or 0.1 mg Se/kg) to high inclusion diets (0.30 to 0.50 mg Se/kg) in growing and finishing pigs. Selenium retention substantially increased, particularly when Se was supplied in the form of Se-Yeast. Increasing SeO₃⁻^2^ only slightly increased Se concentration in the loin. Whereas, Batorska et al. (2017) showed at least 130% increase of Se when fed a diet with Se-yeast compared with SeO₃⁻^2^.

Li et al. (2024) conducted a similar study using nursery pigs and studied the speciation of Se compounds in various tissues. After 28 days of diets supplemented with SeO₃⁻^2^ (0.3 mg/kg) or Se-yeast (0.3 mg/kg) from 9.2 kg body weight, the loin Se concentrations were 0.15 (SeO₃⁻^2^) and 0.35 (Se-Yeast) μg/g, indicating that the pigs with SeMet had 172% more Se in the loin than pigs with SeO₃⁻^2^, in line with the observations in grower-finishers. Interestingly, the large majority of this increase of Se could be attributed to an increase in SeMet which accounted for 85% of the Se species in muscle.

Se concentrations in other tissues, such as the liver, kidney, and hoof, also increased when pigs were fed organic Se compared with the inorganic Se source, and each tissue increased in Se concentration as the dietary Se level increased (Kim and Mahan 2001).

As reviewed in the previous section, these Se body reserves in the form of SeMet can be released due to protein turnover. The SeMet then becomes available for trans-selenation, resulting in an increase of SeCys concentration and consequently in the production of selenoproteins.

## Selenocysteine in antioxidative protection

The activity of selenoproteins is highly dependent on the presence of SeCys, which provides the catalytic efficiency required to neutralize oxidative threats. The ability of SeCys to undergo rapid redox cycles makes it uniquely suited for these roles, giving selenoproteins like GSH-Px superior capacity to scavenge ROS compared to non-Se-containing enzymes (Calamari et al. 2010; Liu et al. 2021b). In pigs under oxidative stress, selenoproteins have been shown to play a vital role in maintaining cellular redox balance (Chen et al. 2012; Li et al. 2021). For example, pigs experiencing oxidative stress due to weaning or rapid growth have benefited from Se supplementation, which supports activity of selenoproteins and enhances resistance to oxidative damage (Gan et al. 2014; Liu et al. 2021c). In addition, Se supplementation improves immune function, reduces inflammation, and supports overall health during periods of stress (Sun et al. 2017; Falk et al. 2018).

### Glutathione peroxidases

Selenium's antioxidant role is most notable in its involvement in GSH-Px, a key component of the body's defense against oxidative damage. The GSH-Px helps mitigate oxidative stress by catalyzing the reduction of organic hydroperoxides to their corresponding alcohols. The GSH-Px forms a homotetramer in which each of the 4 separate subunits contains Se as SeCys at its reactive site. In mammals, there are several GSH-Px paralogs containing a SeCys residue at their active site. GSH-Px1 is a ubiquitous enzyme expressed in all cell types, with the highest expression levels observed in the liver and kidney. In contrast, the expression of GSH-Px2 and GSH-Px3 is limited to certain tissues. GSH-Px2 is primarily found in the epithelium of the gastrointestinal tract, whereas GSH-Px3 is secreted primarily from the kidney and is the major GSH-Px form in plasma (Labunskyy et al. 2014). The GSH-Px3 helps protect extracellular environments from oxidative stress (Burk et al. 2001; Steinbrenner and Sies 2009).

### Thioredoxin reductases

Thioredoxin reductases are another class of selenoproteins involved in preventing oxidative stress and regenerating reduced thioredoxin (Trx). Thioredoxins are themselves small redox proteins that play an essential role in redox signaling and the reduction of oxidized components, such as Cys-Cys disulfide bonds, protecting cells and tissues from oxidative stress (Labunskyy et al. 2014). Similar to the GSH system, Trx and TrxR make up the thioredoxin system and are involved in multiple ROS scavenging processes through reversible Trx oxidation/reduction reactions catalyzed by TrxR. A SeCys residue is present at the active site. Three TrxR isozymes exist: thioredoxin reductase 1 and 2 are ubiquitous, located respectively in the cytosol and in the mitochondria. Thioredoxin reductase 3 is located in the testis.

### Selenoglutathione

Selenoglutathione (GSeH) plays a critical role in the antioxidative defense system by scavenging free radicals and reducing oxidative stress (Weaver and Skouta 2022). As a Se-containing analog of GSH, GSeH provides enhanced protection due to the unique properties of Se, which can efficiently catalyze redox reactions (Lee and Roh 2023). SeCys, as part of GSeH, contributes directly to the higher reactivity and the higher efficiency of redox reactions, enabling more efficient detoxification of peroxides and free radicals compared to its sulfur-containing counterpart, GSH (Weaver and Skouta 2022).

Under disease states or oxidative stress conditions, such as late gestation or early lactation, as well as after weaning animals experience increased oxidative damage (Duarte et al. 2019; Zhao and Kim 2020). During these periods, the demand for Se may rise, as Se is essential for the regeneration and function of antioxidant systems, including GSeH. Supplementing animals with organic Se sources, like SeCys or SeMet, has been shown to improve antioxidative capacity by enhancing the production of selenoglutathione and reducing oxidative damage in tissues (Liu et al. 2021b).

## Application of selenium-containing amino acids for pigs under oxidative stress

Next to the basal level of feed raw materials, Se is currently fed to farm animals, either in an inorganic or organic form, to prevent Se deficiency and mitigate subsequent oxidative stress. The inorganic forms are mainly mineral salts, such as SeO₃⁻^2^ or selenate (SeO^2^⁻₄). Organic forms of Se are typically obtained from microorganisms such as bacteria and yeast from their metabolic process of synthesizing methionine and cysteine (Schrauzer 2000; Papp et al. 2007; Krittaphol et al. 2011; Lima et al. 2018). When Se is available at the time of biosynthesis of methionine and cysteine, these microorganisms could use Se instead of sulfur resulting in the production of SeMet and SeCys. To obtain SeMet and SeCys, yeast is predominantly used in large-scale fermentation produced in concentrated or purified forms. These Se sources are collectively called ‘Se-enriched yeast’ and have been extensively used in pig production (Mahan et al. 1999; Mateo et al. 2007; Rao et al. 2023), whereas their efficacy is affected (1) by the concentrations of Se-containing amino acids compared to remaining inorganic Se and also (2) by the ratio between SeMet and SeCys due to the unique differences in their functions. The analysis from 15 Se-enriched yeast sources showed that an average of 65% and 17% Se were SeMet and SeCys, respectively, whereas 3% Se was inorganic Se and the remaining 16% was unidentified organic Se (EFSA 2016). This indicates that Se-enriched yeast provides biologically and nutritionally significant amounts of SeMet and SeCys if supplemented in pig diets.

There is a limited number of research papers demonstrating the efficacy of Se-containing amino acids in pigs under oxidative stress. Available research has been focused on the role of SeMet for its antioxidative function compared to inorganic Se (SeO₃⁻^2^). Literature regarding the direct dietary application of pure or free SeCys in pigs is currently limited or unavailable. Due to its high chemical reactivity and rapid oxidizing property (Arnér, 2010; Chung and Krahn 2022), it is not practical to produce free SeCys supplement for feed application. Table 2 lists published papers showing the efficacy of SeMet when fed to pigs in relation to their oxidative stress status. Most applications of organic forms of Se in pig diets was the use of Se-enriched yeast, which is rather practical and affordable source of Se-containing amino acids. These will be reviewed in the next section.

**Table 2:** Oxidative stress status of pigs fed diets with different Se sources

Reference	Se source and inclusion (mg/kg)	Body weight (kg) Feeding duration (d)	Oxidative stress status (% changes)
(Cao et al. 2014)	SeO₃⁻^2^ (0.3) Selenomethionine (0.1–0.7)	9.9 to 25 kg 42 d	Serum GSH-Px (+11%) Muscle GSH-Px (+47%)
(Jing et al. 2023a)	Selenomethione (0.0–0.9)	25 kg 112 d	Muscle GSH-Px (+198%) Muscle ROS (−17%)
(Liu et al. 2021a)	Selenomethionine (0–0.6)	50 kg BW 28 d	Serum GSH-Px (+66%) Muscle GSH-Px (+55%)
(Zhang et al. 2022)	Selenomethionine (0–0.5)	125 d-old pigs 30d	Serum GSH-Px (+60%)

Cao et al. (2014) used newly weaned pigs (at 28 day of age) fed diets with SeO₃⁻^2^ (0.3 mg/kg), increasing levels of SeMet (0.1 to 0.7 mg/kg), and no added Se (control) for 42 days until reaching 25 kg body weight. Addition of Se reduced oxidative damage product (MDA) in the liver and muscle regardless of its sources. However, use of SeO₃⁻^2^ did not increase antioxidative capacity as it did not increase the production of GSH-PX. However, the use of SeMet at 0.1 to 0.3 mg/kg increased antioxidative capacity of serum, liver, and muscle by increasing the production of GSH-PX indicating effectiveness of SeMet reducing oxidative damages to tissues and also increasing antioxidative capacity compared to SeO₃⁻^2^.

Similarly, Jing et al. (2023a) used pigs at 25 kg body weight fed a diet without Se or oxidized diets with increasing levels of SeMet (0 to 0.9 mg/kg) for 16 weeks. Feeding oxidized diet without Se reduced serum Se compared to a normal diet without Se. Supplementing SeMet to oxidized diet increased Se concentration in the serum and muscle. Feeding oxidized diet did not increase oxidative damage products in the muscle but increased the ROS concentration and reduced antioxidative capacity. Supplementation of SeMet reduced the ROS concentration and increased GSH-PX activities without affecting antioxidative capacity in the muscle. Supplementing SeMet increased the expression of genes for various selenoproteins in the muscle, reducing oxidative damage from consuming oxidized diets.

Liu et al. (2021b) used pigs at 50 kg body weight fed a diet without Se at normal ambient temperature (22 °C) or diets with increasing levels of SeMet (0 to 0.6 mg/kg) at chronic heat stress condition (33 °C) for 28 days. Heat stress condition increases oxidative stress in pigs (Zhao and Kim 2020). Chronic heat stress condition did not affect Se concentrations in the serum and muscle whereas supplementation of SeMet increased them. Chronic heat stress condition increased oxidative damage (MDA) both in the serum and the muscle, whereas the supplementation of SeMet reduced it by increasing the GSH-Px activity in the serum and muscle and the expression of genes for GSH-Px4 in the muscle. Zhang et al. (2022) used 125 day old pigs and half of them were exposed to ammonia (90 mg/m^3^ intended to induce intestinal barrier dysfunction). Dietary treatment was SeMet supplementation (0 or 0.5 mg/kg; fed for 30 days) to evaluate its role in protecting the intestinal barrier function. Exposing pigs to ammonia increased blood ammonia and reduced Se concentration in the intestinal tissue, whereas the supplementation of SeMet reduced blood ammonia and increased Se concentration in the intestinal tissue resulting in increased GSH-Px activity, reduced ROS concentration, reduced oxidative damages (MDA), and increased expression of genes for tight junction proteins in the intestinal tissue. Falk et al. (2020) used sows from their late gestation (1 month before farrowing) to the end of lactation (34 days after farrowing) fed diets with SeO₃⁻^2^ (0.4 and 0.6 mg/kg for gestation and lactation, respectively) or SeMet (0.26 and 0.43 mg/kg for gestation and lactation, respectively). Piglets were investigated for their Se availability, oxidative stress status, and antioxidative capacity until 38 day of age. Piglets from sows with SeMet had higher plasma Se and SeMet concentrations during lactation compared to piglets from sows with SeO₃⁻^2^ resulting in increased GSH-Px in the plasma of piglets.

Similar research evidences showing the effects of SeMet reducing oxidative stress can be found in other animal species including lactating dairy cow (Sun et al. 2019), beef cattle (Liao et al. 2011), rabbits (Liu et al. 2021d), mice (Staneviciene et al. 2023), laying hens (Laika and Jahanian 2015; Dos Reis et al. 2019), and broiler chickens (Jing et al. 2023b). The benefits of the use of SeMet in feeding pigs (as well as other animal species) under oxidative stress are rather clear compared with the use of SeO₃⁻^2^. Considering different biological roles between SeCys and SeMet, it can be hypothesized that the feed application of SeCys in pigs should provide advanced benefits compared to the use of SeMet warranting research investigations. However, the need of SeCys from dietary sources is not considered significant if the body reserves sufficient SeMet that can be released and converted to SeCys (Fig. 4; Esaki et al. 1981; Seale 2019; Bierla et al. 2023). Dietary SeCys is effectively metabolized to hydrogen selenide in the liver for the de novo SeCys synthesis for the function as a part of selenoproteins and thus continuous supply of SeCys is rather important than high provision through dietary sources. Considering that 17% of Se in Se-enriched yeast is SeCys, dietary supplementation of Se-enriched yeast would provide sufficient SeCys to pigs, whilst SeMet (65% Se) would be stored in body proteins and continuously provide Se for the synthesis of SeCys upon needs. Under oxidative stress, the use of body Se would prioritize to the synthesis of SeCys and GSeH by increasing the expression of *GSeH* (Mirault et al. 1991; Yagi et al. 1996).

## Application of Se-enriched yeast for pigs under oxidative stress

Supplementation of Se through Se-enriched yeast has been shown to be effective in a dose-dependent manner regardless of Se adequacy. Zhou et al. (2009) used nursery pigs fed Se-deficient diets (0.02 mg Se/kg) with gradual increase of Se-enriched yeast to provide up to 3.0 mg Se/kg for 8 weeks. Dietary supplementation of Se-enriched yeast increased hepatic Se concentrations and GSH-Px activities in a dose-dependent manner indicating the efficacy of Se-enriched yeast for its antioxidative function. Mateo et al. (2007) used growing-finishing pigs fed Se-adequate diets (1.8 mg Se/kg) with gradual increase of Se-enriched yeast to provide up to 5.4 mg Se/kg for 16 weeks until pigs reach market weights (130 kg). Dietary supplementation of Se-enriched yeast increased Se concentrations in the liver, loin, and hair and improved the shelf life of the loin by reducing drip loss indicating the efficacy of Se-enriched yeast for its antioxidative function.

### Oxidative stress and immune challenge models

There are strong research evidences showing the effectiveness of the use of Se-enriched yeast providing antioxidative protection to pigs under oxidative stress conditions. In animal models, the administration of diquat (1,1'-Ethylene-2,2'-bipyridylium dibromide) is used to generate acute and controlled oxidative stress by enhancing the production of ROS species in the body. Doan et al. (2020) used nursery pigs at 9.7 kg body weight fed a Se-deficient diet or diets with Se-enriched yeast for 17 days. Pigs were under acute oxidative stress conditions after injection of diquat on day 11. Dietary supplementation of Se-enriched yeast increased endogenous antioxidant activity in various aspects compared with the unsupplemented challenged pigs or pigs supplemented with SeO₃⁻^2^: Noticeably serum GSH-Px and SOD activities as well as hepatic GSH-Px activity were improved helping to fight against diquat-induced injuries, particularly metabolic perturbation in the liver. In addition, it enhanced resilience to the induced inflammation.

Liu et al. (2021b) used nursery pigs at 7.3 kg body weight fed diets with either SeO₃⁻^2^ or Se-enriched yeast for 21 days. Pigs were under acute oxidative stress conditions after injection of diquat on day 16. Dietary supplementation of Se-enriched yeast reduced the expression of genes for pro-inflammatory cytokines (including TNF-α, IL-1β, and IL-6) and increased serum and hepatic levels of CAT, GSH-PX and SOD activities resulting in a total antioxidant capacity associated with a reduction of MDA concentration. Diquat-induced damage in the liver and kidneys were also reduced.

These studies provide research evidence indicating the efficacy of Se-enriched yeast for its antioxidative function in pigs under oxidative stress conditions which was associated with improved oxidative balance and suppressed inflammatory response. It contributed to improve feed intake, apparent nutrient digestibility, and alleviating growth retardation or reduced feed efficiency (Doan et al. 2020; Liu et al. 2021b).

Immune challenge models have also been used to create conditions to induce oxidative stress. Royer et al. (2016) used nursery pigs at 8.7 kg body weight fed a control diet or diets with Se-enriched yeast and vitamin E for 40 days. Pigs were under stress conditions from multiple vaccinations and high ambient temperature at 37 °C over 6 h period during 2 days in each week. Dietary supplementation of Se-enriched yeast and vitamin E increased serum GSH-Px activities. Lv et al. (2020) used nursery pigs at 6.4 kg body weight fed diets with either SeO₃⁻^2^ or Se-enriched yeast for 27 days. Pigs were challenged with *Salmonella typhimurium*. Dietary supplementation of Se-enriched yeast also increased serum GSH-Px activities compared to SeO₃⁻^2^. These studies provide research evidence indicating the efficacy of Se-enriched yeast for its antioxidative function in pigs under stress and disease challenge conditions.

### Application to growing-finishing pigs for meat quality

Lipid peroxidation induced by free radicals attacks polyunsaturated fatty acids in muscle, destroying the integrity of the muscle cell membrane, which is one of the leading causes of the loss of intracellular fluids (Chen et al. 2019). In addition, the interactions between myoglobin and oxidized lipid are widely accepted to jointly govern meat color, and the autoxidation of myoglobin is the main reason for deviations from bright cherry-red to brown (Ramanathan et al. 2020). Se-enriched yeast has been used in feeds for growing and finishing pigs to enhance the antioxidative capacity of the muscle to improve the meat quality and shelf-life of pork (Table 3). Miqueletto et al. (2025) has shown that pigs supplemented with 0.3 mg/kg organic Se that contains 2/3 SeMet and 1/3 SeCys produced by *Saccharomyces cerevisiae* yeast for 50 days during the finishing phase had a 3-fold increase of Se accretion in the meat as compared to the control. This resulted in improved meat quality including tenderness and maintaining color stability during a 28-day vacuum packed storage at 4 °C. Drip loss is particularly important for pork (Pearce et al. 2011) and it is also indicative of cooking losses. Several studies reported that organic Se is superior to inorganic Se in reducing water loss and improving meat color.

**Table 3:** Meat quality measurement of pigs fed diets with different Se sources

Reference	Se source and inclusion (mg/kg)	Body weight (kg) Feeding duration (d)	Meat quality measurement (% changes)
(Chen et al. 2019)	Se-enriched yeast (0.0) Se-enriched yeast (0.3)	75 kg 45d	(Drip loss) Se-enriched yeast (−51%)
(Mateo et al. 2007)	SeO₃⁻^2^ (0.3) Se-enriched yeast (0.3)	34 kg 110d	(Drip loss) Se-enriched yeast (−13%)
(Zhang et al. 2020)	SeO₃⁻^2^ (0.3) Se-enriched yeast (0.3) Selenomethionine (0.3) SS + Se-Met (0.15 + 0.15)	110–160 kg 50 d	(Backfat, drip loss) Se-enriched yeast (+34%, −11%) Selenomethionine (0%, + 21%) SS + Se-Met (+12%, −20%)

The ability of muscle tissue to retain water is often measured by drip loss. If the value is too high, a large amount of water-soluble protein and sarcoplasmic proteins will be lost, which will seriously affect the color, flavor, and tenderness (Mikulski et al. 2009). Chen et al. (2019) used finishing pigs at 75 kg body weight, fed a control diet or a diet with Se-enriched yeast (0.30 mg Se/kg) for 45 days. Dietary supplementation of Se-enriched yeast increased Se concentration and SOD activity in the loin with reduced MDA concentration. Mateo et al. (2007) used growing pigs at 34 kg body weight, fed a diet with SeO₃⁻^2^ (0.3 mg Se/kg) or a diet with Se-enriched yeast (up to 0.3 mg Se/kg) for 110 days until reaching market weight. Dietary supplementation of Se-enriched yeast increased Se concentration in the loin and serum, resulting in reduced loin drip loss compared to SeO₃⁻^2^. Zhang et al. (2020) used growing crossbred pigs aged 110 days, fed a diet with SeO₃⁻^2^ (0.3 mg Se/kg) or a diet with Se-enriched yeast (up to 0.3 mg Se/kg) for 50 days. Dietary supplementation of Se-enriched yeast reduced drip losses and improved meat color (with decreased L and b values). A study by Calvo et al. (2017) showed that dietary supplementation with Se-enriched yeast improves the meat color of fattening pigs. Meanwhile, a significant increase in the redness (a ∗) and lightness (L ∗) of pork after slaughter and 7 days of cold storage was observed.

These studies provide research evidence indicating the efficacy of Se-enriched yeast in enhancing the antioxidative capacity of meat and reducing markers of oxidation, thereby improving meat quality, particularly reducing drip loss and improving meat color.

### Application to sows for reproductive outcomes

Sows are under elevated oxidative stress conditions with reduced antioxidant capacity, especially during late gestation and lactation (Berchieri‐Ronchi et al. 2010; Weaver and Kim 2014). Oxidative stress is exacerbated by hyperprolificacy due to the greater energy requirements for fetal growth during gestation (Lee and Roh 2023). Serum Se in sows begins to decline from 60 days of gestation and reaches the lowest concentration near parturition. Simultaneously, GSH-Px activity also declines (Mahan et al. 2005), and increased oxidative damage in sows during late gestation may negatively affect the growth and health of fetuses and the postpartum growth of piglets (Zhao et al. 2013). After birth, since newborn piglets obtain all nutrients from the sow, the sow's absorption of different Se sources and its ability to transfer Se and related antioxidants to the offspring affect reproductive outcomes of sows and piglet development.

Zhou et al. (2021) performed a meta-analysis to assess the benefits of organic Se compared to inorganic Se, mainly SeO₃⁻^2^, for improving sow reproductive performance and piglet development. A total of 19 studies were included in the work (Mahan and Kim 1996; Mahan 2000; Mahan and Peters 2004; Yoon and McMillan 2006; Quesnel et al. 2008; Svoboda et al. 2009; Ma et al. 2014; Falk et al. 2020; Mou et al. 2020; Li et al. 2021). In most studies, Se-enriched yeast was used as the organic source of Se. For the remaining studies, pure SeMet (the form in which at least 70% of Se from yeast originates) was used. It was shown that feeding organic Se (0.2 to 0.5 mg/kg) increased the Se concentration in serum (7.7%), colostrum (44.8%), and milk (69.5%), reflecting increased bioavailability of Se from Se-enriched yeast. The meta-analysis showed that Se from an organic source was successfully transferred to piglets, as evidenced by 29.4% increased serum Se concentration and a 6.4% increase in GSH-Px activity in piglets from sows consuming diets supplemented with organic Se.

Looking at antioxidative balance more in detail, Chen et al. (2016) made a comprehensive study on oxidative stress and antioxidative markers of sows fed Se-enriched yeast (0.30 mg Se/kg) during the full gestation and lactation. The Se-enriched yeast enhanced the serum antioxidative capacity associated with higher levels of anti-oxidative enzymes (GSH-Px, SOD), and GSH levels together with a decreased MDA concentration. Colostrum and milk also showed increased Se levels, anti-oxidative capacity, antioxidant enzymes, GSH and decreased MDA concentration. The quality of milk was improved as well: Total solids, solids-not-fat, protein, and lactose levels in milk were greater but similar in colostrum, when sows were fed organic Se.

## Conclusion

Oxidative stress poses a serious challenge to the health and productivity of pigs at different life stages. Upon weaning, dietary and disease challenges cause intestinal immune reactions resulting in an inflammatory reaction and oxidative stress leading to oxidative damage of intestinal tissues. In finishing pigs, oxidative stress compromises growth, intestinal health, and meat quality. For sows, oxidative stress impacts reproductive efficiency, lactation, and immune function, whereas in boars, it leads to decreased semen quality and overall reproductive performance. The consequences of oxidative stress are wide-reaching, but by adopting preventive measures, such as improving the antioxidant concentration of feed, producers can mitigate its negative effects. Various types of antioxidants are used in feed as supplements with similar or different mechanisms. Early intervention and proactive management are key to reducing oxidative stress and enhancing the health, well-being, and productivity of pigs. Selenium is a vital essential micronutrient necessary to build an efficient antioxidative system: serving as an integral component of selenoproteins such as GSH-Px, or can boost effectiveness of co-factors. These selenoenzymes and co-factors are essential for neutralizing reactive oxygen species, mitigating oxidative damage, and preserving cellular homeostasis.

Selenium-containing amino acids are unique source of Se with antioxidative functions as a part of selenoproteins. Selenium-containing amino acids include SeMet and SeCys that can be incorporated into body proteins, with unique metabolic pathways and functions. Whilst SeMet is a non-specific reservoir for Se storage, available through protein degradation, SeCys is at the active site of the selenoprotein production, the central players of the antioxidant system, and its demand can dramatically increase in oxidative stress conditions. During this demand, the Se reserve deposited in the form of SeMet will be mobilized and increases the level of SeCys to enhance the antioxidant effect of Selenium enriched yeast. There are research evidences showing the effectiveness of the use of Se-enriched yeast in reducing oxidative stress in pigs compared to the use of SeO₃⁻^2^. Se-enriched yeast is a natural source of organic Se, mainly available in the form of SeMet on the one hand and SeCys and other Se compounds on the other hand. Se-enriched yeast shows higher bioavailability compared to inorganic sources. With several decades of use in animal supplements, Se-enriched yeast have also proven their superior efficacy at sustaining a better anti-oxidant status of various categories of pigs as compared to inorganic Se, improving overall health and enhancing performance and pork quality.

## Data Availability

All cited papers in this review are publicly available and accessible using doi.

## Abbreviations

H_2_O_2_: Hydrogen peroxide
GSH: Glutathione
GSH-Px: Glutathione peroxidase
MDA: Malondialdehyde
NO: Nitric oxide radical
ROO: Peroxyl radicals
ROS: Reactive oxygen species
Se: Selenium
SeO^2^⁻₄: Selenate
SeCys: Selenocysteine
GSeH: Selenoglutathione
GSSeSG: Selenoglutathione disulfide
SEPHS2: Selenophospahte synthetase 2
SeO₃⁻^2^: Sodium selenite
O_2_^•^^−^: Superoxide
SOD: Superoxide dismutase
SECIS: Selenocysteine insertion sequence
